# Imaging and biomarker-based risk stratification in TAVI: the role of epicardial fat, visceral fat, and adiponectin

**DOI:** 10.1093/ehjopen/oeag085

**Published:** 2026-05-29

**Authors:** Tatyana Storozhenko, Dimitri Buytaert, Raffaella Mistrulli, Michele Mattia Viscusi, Sara Corradetti, Emiliano Fiori, Leen Delrue, Monika Beles, Jozef Bartunek, Liesbeth Rosseel, Eric Wyffels, Marc Vanderheyden

**Affiliations:** Cardiovascular Center Aalst, Moorselbaan 164, Aalst 9300, Belgium; Department of Prevention and Treatment of Emergency Conditions, L.T. Malaya Therapy National Institute NAMSU, 2a Lyubovi Maloy Avenue, Kharkiv 61039, Ukraine; Cardiovascular Center Aalst, Moorselbaan 164, Aalst 9300, Belgium; Cardiovascular Center Aalst, Moorselbaan 164, Aalst 9300, Belgium; Department of Clinical and Molecular Medicine, Sapienza University, Via Giorgio Nicola Papanicolau, Rome 00189, Italy; Cardiovascular Center Aalst, Moorselbaan 164, Aalst 9300, Belgium; Department of Advanced Biomedical Sciences, University Federico II, Via Sergio Pansini 5, Naples 80131, Italy; Cardiovascular Center Aalst, Moorselbaan 164, Aalst 9300, Belgium; Department of Clinical and Molecular Medicine, Sapienza University, Via Giorgio Nicola Papanicolau, Rome 00189, Italy; Cardiovascular Center Aalst, Moorselbaan 164, Aalst 9300, Belgium; Department of Clinical and Molecular Medicine, Sapienza University, Via Giorgio Nicola Papanicolau, Rome 00189, Italy; Cardiovascular Center Aalst, Moorselbaan 164, Aalst 9300, Belgium; Cardiovascular Center Aalst, Moorselbaan 164, Aalst 9300, Belgium; Cardiovascular Center Aalst, Moorselbaan 164, Aalst 9300, Belgium; Cardiovascular Center Aalst, Moorselbaan 164, Aalst 9300, Belgium; Cardiovascular Center Aalst, Moorselbaan 164, Aalst 9300, Belgium; Cardiovascular Center Aalst, Moorselbaan 164, Aalst 9300, Belgium

**Keywords:** Transcatheter aortic valve implantation, Epicardial adipose tissue, Visceral adipose tissue, Adiponectin, Outcomes

## Abstract

**Aims:**

Transcatheter aortic valve implantation (TAVI) has become a standard treatment for severe aortic stenosis, yet clinical outcomes vary significantly. Epicardial adipose tissue (EAT), visceral adipose tissue (VAT), and circulating adiponectin may contribute to this heterogeneity. This study aimed to determine how adipose tissue characteristics, in combination with adiponectin levels, affect clinical outcomes following TAVI.

**Methods and results:**

This single-centre retrospective study included 243 patients with severe aortic stenosis who underwent TAVI. Pre-procedural computed tomography scans were processed using artificial intelligence to quantify EAT density (EAT_HU_) and VAT area at the level of the L3 vertebra (VAT_L3A_). Patients were stratified into groups based on median adiponectin (6.54 µg/mL), VAT_L3A_ (135 mm), EAT_HU_ (−82.0 HU), and EAT_VOL_ (31 mL). The primary endpoint was 1-year major adverse cardiac and cerebrovascular events (MACCE), defined as all-cause mortality, heart failure hospitalization, or stroke. Over a mean follow-up of 344 ± 77 days, there were 25 deaths (10%), 12 heart failure hospitalizations (5%), and 7 strokes (3%). Circulating adiponectin (adjusted HR 1.34 per 5 μg/mL, 95% CI 1.05–1.70; *P* = 0.017) and VAT_L3A_ (adjusted HR 0.63 per 100 mm^2^, 95% CI 0.44–0.91; *P* = 0.014) independently associated with MACCE, EAT_HU_, and EAT_VOL_ did not. All three combined adipose–adiponectin phenotypes demonstrated significant graded risk (*P*-for-trend 0.003–0.048), with the high EAT_VOL_/high adiponectin phenotype showing the strongest association (adjusted HR 4.25, 95% CI 1.80–10.07).

**Conclusion:**

Circulating adiponectin and VAT_L3A_ independently predict 1-year MACCE after TAVI. EAT parameters become prognostically informative only when combined with adiponectin.

## Graphical Abstract

**Graphical Abstract:**
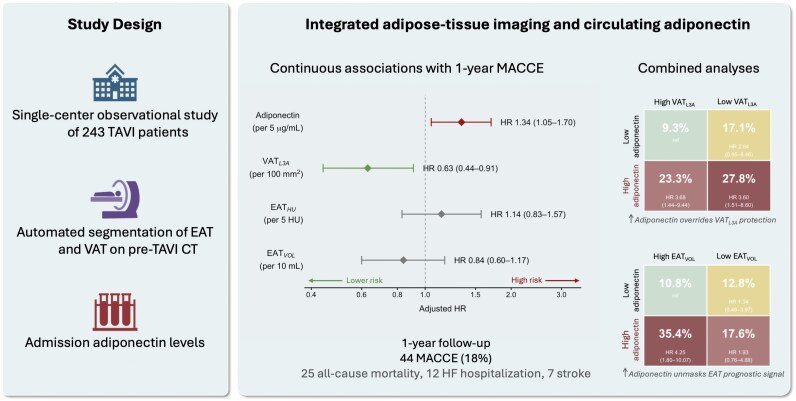

## Introduction

Transcatheter aortic valve implantation (TAVI) has become a well-established alternative to surgical aortic valve replacement for patients with severe aortic stenosis (AS).^1–5^ As the indications for TAVI expand and its use becomes more widespread, there is increasing emphasis to refine risk stratification and optimize patient selection to improve clinical outcomes. Traditional risk assessment models, such as the Society of Thoracic Surgeons (STS) score and EuroSCORE II, primarily focus on demographic and clinical variables. However, these tools often fail to capture the complex metabolic and inflammatory processes that drive aortic valve degeneration and influence recovery after intervention.^6^

An ‘obesity paradox’ has been described in TAVI with several studies reporting better survival among overweight and obese patients.^7,8^ This observation highlights the limitations of body mass index (BMI) as a marker of cardiovascular risk and underscores the importance of regional adipose tissue depots.^9,10^ Two computed tomography (CT)-derived adipose compartments accessible from the routine pre-procedural work-up offer more biologically specific information: Epicardial adipose tissue (EAT), a metabolically active fat layer in close proximity to the myocardium, and visceral adipose tissue (VAT).^11–13^ Both EAT and VAT demonstrated associations with adverse outcomes after TAVI.^14–19^ Despite this, findings remain inconsistent across cohorts, particularly regarding whether fat attenuation or volume provides greater prognostic value. This heterogeneity suggests that the prognostic significance of adipose tissue characteristics may depend on the underlying metabolic and neurohormonal state.

In this context, adiponectin, an adipokine secreted predominantly by adipocytes, represents a potential link between adipose tissue biology and clinical outcomes. Although traditionally regarded as anti-inflammatory and cardioprotective,^20,21^ elevated adiponectin levels have been paradoxically associated with poor prognosis in older adults after TAVI.^22–24^ Yet the combined prognostic value of EAT, VAT, and adiponectin has not been systematically explored in the TAVI population.

Accordingly, this study aimed to investigate the prognostic significance of CT-derived EAT and VAT characteristics, in combination with circulating adiponectin levels, for 1-year outcomes after TAVI.

## Methods

### Study design and population

This retrospective, single-centre, observational study included consecutive patients with severe symptomatic AS who underwent TAVI between June 2021 and September 2024 at the Cardiovascular Center Aalst, Aalst, Belgium. Patients were included if pre-procedural cardiac CT imaging and stored serum samples for adiponectin assessment were available. The study flow diagram is illustrated in Supplementary material online, *Figure S1*. All patients were managed according to current European Society of Cardiology guidelines, and the indication for percutaneous aortic valve replacement was determined by the local Heart Team.^25^ The present study was conducted according to the principles outlined in the Declaration of Helsinki. All patients were informed about their participation in the study and provided informed consent for the anonymous publication of scientific data. The data that support the findings of this study are available from the corresponding author upon reasonable request.

### Quantification of adipose tissue using CT

**Figure 1:**
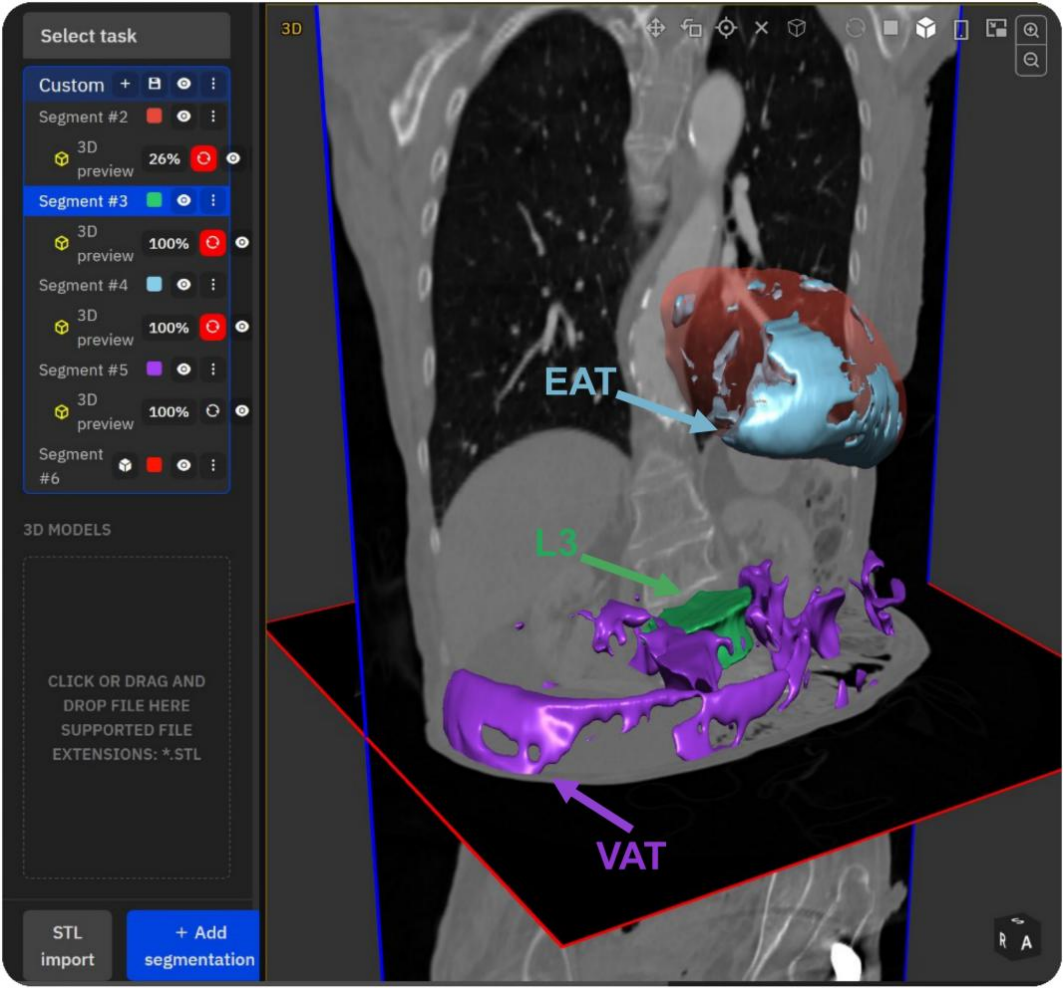
3D CT-based segmentation of adipose tissue and anatomical landmarks in a patient undergoing TAVI. EAT (cyan) is visualized surrounding the myocardium within the pericardial sac. VAT (purple) is segmented at the level of the L3, shown in green. The segmentation was performed using a semi-automated pipeline to quantify adipose compartments. These volumetric and density-based measurements (EAT_HU_ and VAT_L3A_) were used for phenotypic risk stratification in the study. CT, computed tomography; EAT, epicardial adipose tissue; HU, Hounsfield units; L3A, lumbar vertebra 3 area; VAT, visceral adipose tissue; TAVI, transcatheter aortic valve implantation.

Pre-TAVI CT was performed for procedural planning using a standardized acquisition protocol consistent with Society of Cardiovascular Computed Tomography recommendations.^26^ A custom artificial intelligence (AI) pipeline, developed and implemented in Medannot, an AI-driven collaborative web platform for radiological annotation and 3D (Medannot, Bratislava, Slovakia), was utilized to quantify EAT (*Figure 1*). EAT was defined as the adipose tissue located between the myocardium and the pericardium. Initially, a pre-trained nnU-Net model was employed to segment the pericardial sac. Training annotations were derived from an independent expert-annotated cardiac CT cohort, for which the annotation methodology has been previously described.^27,28^ Internal 5-fold cross-validation yielded a mean held-out Dice of 0.92. For each patient in the present cohort, the inferred pericardial-sac mask was opened on the platform, visually inspected by a trained operator, and downloaded. The model output was consistently satisfactory, and no manual adjustments were applied. Subsequently, a threshold Hounsfield unit (HU) range between −195 and −45 HU corresponding to the density of fat was applied to the segmented region.^13^ The total volume and average HU of the resultant region, denoted as EAT_VOL_ and EAT_HU_, respectively, were calculated, with EAT_HU_ reflecting the tissue composition (lipid-rich vs. fibrotic or inflamed tissue). Cohort-specific median thresholds were used to stratify patients into high and low groups for each CT-derived adipose tissue parameter: −82 HU for EAT_HU_, 31 mL for EAT_VOL_, and 135 mm^2^ for VAT_L3A_. To evaluate VAT, TotalSegmentator was used to segment VAT alongside the L3 vertebral body.^29^ The mean VAT area was quantified at the level of the L3 vertebral body (VAT_L3A_) with this axial slice-based measurement serving as a surrogate for total visceral fat burden.^10^

### Laboratory analysis

Peripheral blood samples were collected pre-procedure and stored at −80°C until analysis. Serum adiponectin concentrations were measured using the Human HMW Adiponectin/Acrp30 Quantikine ELISA Kit (R&D Systems, Wiesbaden, Germany). All samples were measured in duplicate, and the mean value was used for analysis. The intra- and inter-assay coefficients of variation were <10%. Serum adiponectin was dichotomized at the cohort median (6.54 µg/mL) to define low and high adiponectin groups.

### Study endpoints

The primary endpoint was major adverse cardiac and cerebrovascular events (MACCE), defined as a composite of all-cause mortality, HF hospitalization, or stroke, within 1 year after TAVI. The secondary endpoint was 1-year all-cause mortality. Clinical outcomes were recorded in adherence to the criteria established by the Valve Academic Research Consortium 3.^30^ Follow-up data were obtained through routine outpatient clinic visits or hospital discharge summaries.

### Statistical analysis

Continuous variables are presented as mean ± standard deviation if normally distributed and as median with interquartile range (IQR) if non-normally distributed. Group comparisons were performed using one-way ANOVA or Kruskal–Wallis test. Categorical variables are expressed as absolute numbers and percentages and compared using chi-square or Fisher’s exact test as appropriate. Correlations between adipose tissue characteristics and circulating adiponectin levels were evaluated using Spearman’s rank correlation coefficient. Restricted cubic splines with three knots were used to assess the continuous associations between adiponectin, VAT_L3A_, EAT_HU_, EAT_VOL_, and the risk of 1-year MACCE. In addition, each biomarker was modelled as a continuous predictor with hazard ratios (HR) expressed per one standard deviation (SD) increase, analysed across quartiles, and combined median-stratified phenotype groups, with the low-risk group as reference. Cumulative incidence rates for MACCE were estimated using the Kaplan–Meier method and compared using the log-rank test. Cox proportional hazards models were fitted to assess associations between adipose tissue characteristics, adiponectin levels, and 1-year outcomes. The proportional hazards assumption was verified using Schoenfeld residuals. The primary multivariable model was adjusted for age, sex, STS score, and frailty. Two pre-specified sensitivity analyses additionally adjusted for creatinine, atrial fibrillation, and left ventricular ejection fraction (Extended model 1), and further added BMI and NT-proBNP (Extended model 2). To assess potential overfitting, we performed bootstrap internal validation of the Cox models using 200 resamples with the optimism-correction method of Harrell, producing optimism-corrected Harrell’s C-statistics and calibration slopes. Events-per-variable ratios were calculated for all models (see Supplementary material online, *Table S1*). A two-sided *P*-value of <0.05 was considered statistically significant for all statistical tests. Analysis was performed using R software version 4.3.1 (R Foundation for Statistical Computing, Vienna, Austria).

## Results

### Study population

A total of 243 patients were included in the analysis. Baseline characteristics are summarized in *Table 1*. The mean age was 82 ± 6 years, and 54% were female. Patients were stratified into four groups based on the cohort median values of each CT-derived adipose parameter combined with circulating adiponectin, producing three parallel combined phenotype analyses. Baseline characteristics stratified by each phenotype are presented in Supplementary material online, *Tables S2*–*S4*. Among the predefined groups, patients with higher adiponectin levels were older (*P* = 0.001–0.007), more frequently female (*P* < 0.001), and had significantly higher NT-proBNP (*P* < 0.001) and STS score (*P* = 0.002–0.038). In contrast, patients with lower adiponectin levels had higher BMI (*P* < 0.001) and a greater burden of cardiometabolic comorbidities, including hypertension (*P* = 0.003–0.023), dyslipidaemia (*P* = 0.004), and diabetes mellitus (*P* < 0.001–0.033). Importantly, low-adiponectin subgroups, irrespective of EAT characteristics, demonstrated significantly greater VAT_L3A_ values compared to those with higher adiponectin levels (*P* < 0.001).

**Table 1:** Baseline characteristics

	Overall (*n* = 243)
Age, years	81.9 ± 6.2
Female sex	132 (54.3)
BMI, kg/m^2^	25.0 (23.0–28.7)
Diabetes mellitus	75 (30.9)
Hypertension	174 (71.6)
Dyslipidaemia	197 (81.1)
Pre-existing atrial fibrillation	101 (41.5)
NYHA functional Class ≥ II	233 (95.8)
Frailty score	4.0 (4.0–5.0)
STS score	4.3 (2.8–7.0)
LVEF, %	55.4 ± 16.0
AV mean gradient, mmHg	44.2 ± 17.3
AVA, cm^2^	0.75 ± 0.31
Creatinine, mg/dL	1.0 (0.85–1.4)
NT-proBNP, ng/L	1366 (572–2546)
hs-cTn, ng/L	25 (16–53)
Adiponectin, µg/mL	6.54 (3.68–11.77)

Continuous variables are presented as mean ± SD or median (IQR), when appropriate; categorical data as *n* (%).

AV, aortic valve; AVA, aortic valve area; BMI, body mass index; hs-cTn, high-sensitivity cardiac troponin; LVEF, left ventricular ejection fraction; NYHA, New York Heart Association; STS, Society of Thoracic Surgeons.

The median Frailty score of all patients was 4.0 (IQR: 4.0–5.0) with preserved left ventricular ejection fraction (55 ± 16%). Echocardiographic parameters were comparable across groups. The mean aortic valve peak velocity was 4 ± 0.8 m/s, mean transvalvular gradient 44 ± 17 mmHg, and mean aortic valve area 0.75 ± 0.31 cm^2^. All procedures were performed via transfemoral access, predominantly with self-expanding valves (97%). Permanent pacemaker implantation was required in 57 patients (23%), and other major periprocedural complications were infrequent and similar among all groups.

### Correlations between adipose tissue parameters and adiponectin

The distributions of VAT_L3A_, EAT_VOL_, EAT_HU_, and adiponectin in the entire patient cohort are shown in Supplementary material online, *Figure S2*, with the median values of 135.4 (IQR: 81.0–223.3) mm^2^, 31.4 (IQR: 19.0–43.7) mL, −82.0 (IQR: −86.6 to −77.8) HU, and 6.54 (IQR: 3.68–11.77) µg/mL, respectively. EAT_VOL_ correlated strongly and positively with VAT_L3A_ (*r* = 0.71, *P* < 0.001), whereas EAT_HU_ was not associated with VAT_L3A_ (*r* = −0.07, *P* = 0.308). A weak negative correlation was observed between EAT_HU_ and EAT_VOL_ (*r* = −0.27, *P* < 0.001). Adiponectin levels correlated inversely with VAT_L3A_ and EAT_VOL_ (*r* = −0.59 and r = −0.41, respectively, *P* < 0.001 for both), while showing a modest positive correlation with EAT_HU_ (*r* = 0.22, *P* = 0.001) and a moderate positive correlation with NT-proBNP (*r* = 0.33, *P* < 0.001). EAT_HU_ showed a modest positive correlation with IL-6 (*r* = 0.21, *P* = 0.012), NT-proBNP (*r* = 0.22, *P* = 0.006), and hs-cTn (*r* = 0.23, *P* = 0.002). The full correlation matrix is provided in Supplementary material online, *Figure S3*.

### Association between adipose tissue parameters, adiponectin, and clinical outcomes

**Figure 2:**
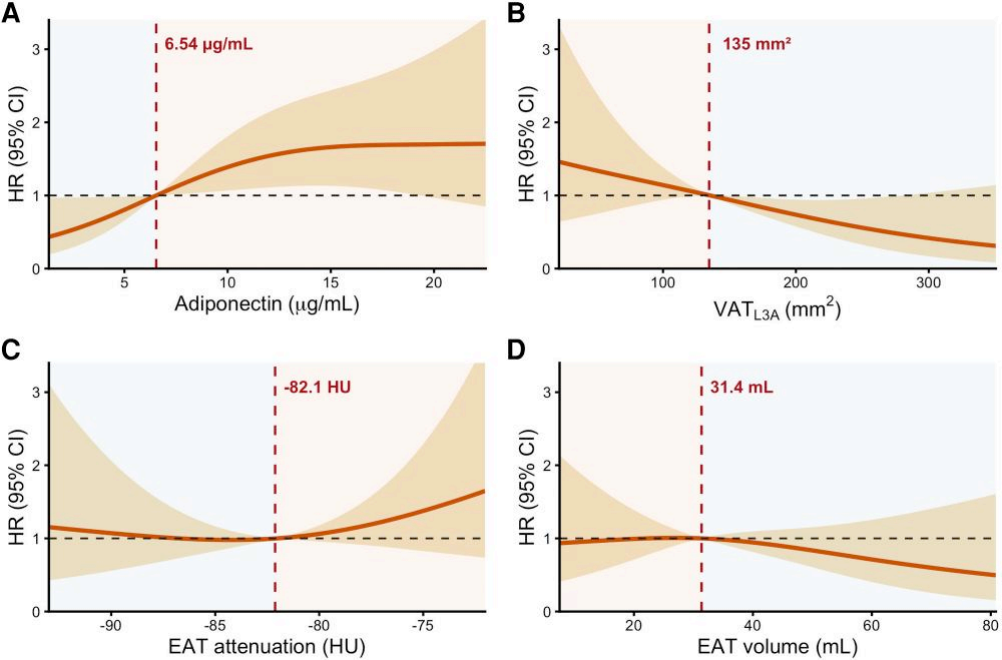
Continuous risk profiling of adipose tissue characteristics and adiponectin for 1-year MACCE after TAVI. HRs are shown as solid lines with shaded areas representing 95% CI and are expressed relative to the median value of each variable. Analyses were multivariable and adjusted for age, sex, STS score, and frailty. CI, confidence interval; EAT, epicardial adipose tissue; HR, hazard ratio; HU, Hounsfield units; L3A, lumbar vertebra 3 area; MACCE, major adverse cardiac and cerebrovascular events; STS, Society of Thoracic Surgeons; TAVI, transcatheter aortic valve implantation; VAT, visceral adipose tissue.

**Figure 3:**
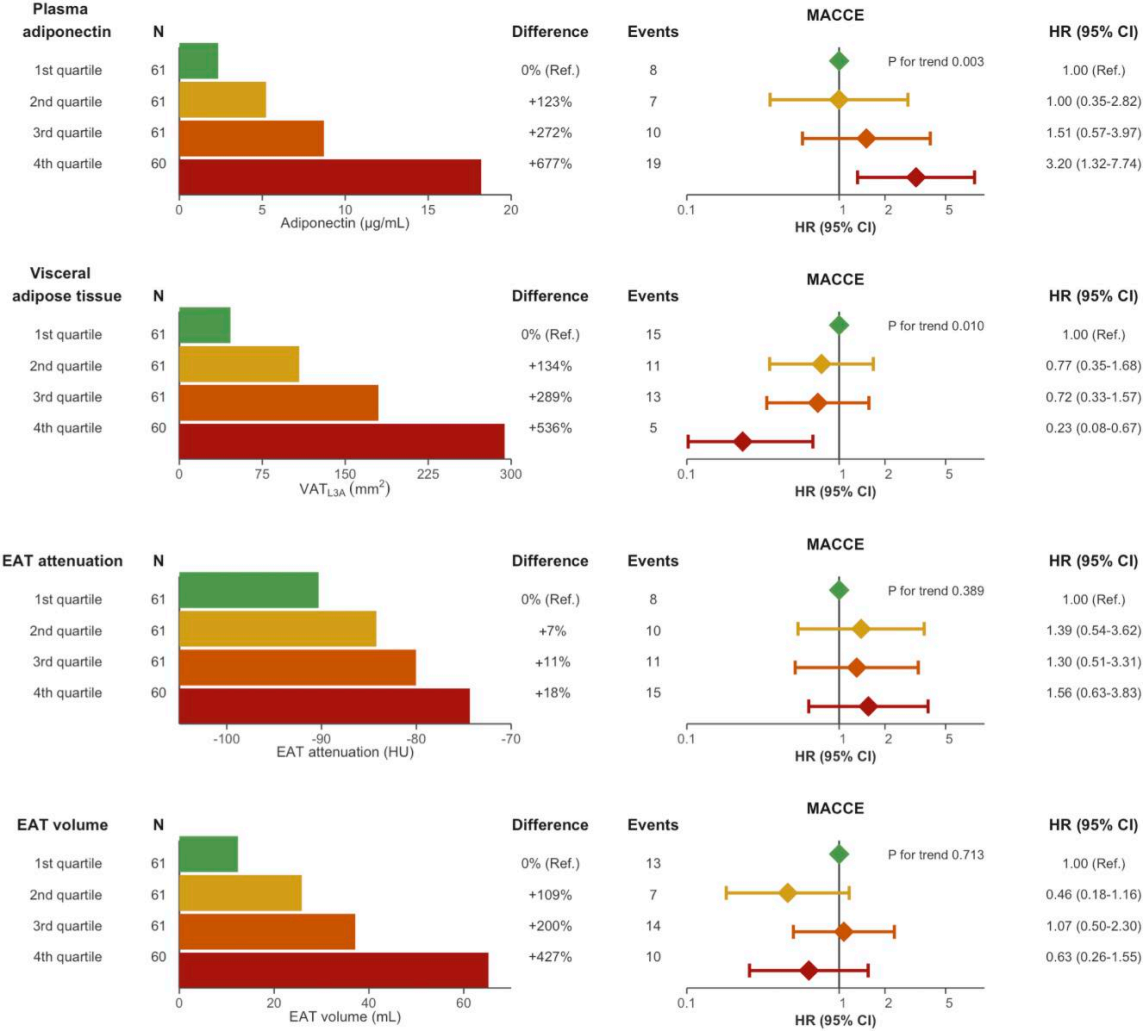
Association of adipose tissue characteristics and adiponectin with 1-year MACCE after TAVI according to quartiles. Mean values of plasma adiponectin, VAT_L3A_, EAT_HU_, and EAT_VOL_ across quartiles are shown as bars. Percentage differences relative to the first quartile are indicated. HRs with 95% confidence intervals for MACCE are indicated with diamonds and whiskers, adjusted for age, sex, STS score, and frailty. CI, confidence interval; EAT, epicardial adipose tissue; HR, hazard ratio; HU, Hounsfield units; L3A, lumbar vertebra 3 area; MACCE, major adverse cardiac and cerebrovascular events; STS, Society of Thoracic Surgeons; TAVI, transcatheter aortic valve implantation; VAT, visceral adipose tissue.

Adiponectin showed a monotonic increase in risk of 1-year MACCE (adjusted HR per 5 μg/mL: 1.34, 95% CI 1.05–1.70; *P* = 0.017). In contrast, higher VAT_L3A_ was associated with a lower risk of adverse outcomes (adjusted HR per 100 mm^2^: 0.63, 95% CI 0.44–0.91; *P* = 0.014). EAT_HU_ and EAT_VOL_ showed no significant associations with MACCE (adjusted HR per 5 HU: 1.14, 95% CI 0.83–1.57; *P* = 0.41; and per 10 mL: 0.84, 95% CI 0.60–1.17; *P* = 0.30, respectively) (*Figure 2*). In the extended multivariable models, the direction of the associations was preserved, although the effect sizes were attenuated (see Supplementary material online, *Figure S4*). Quartile-based models confirmed the patterns observed in spline analyses and identified the lowest-risk reference category for each biomarker (*Figure 3* and Supplementary material online, *Figure S5*).

### Risk stratification by combined adipose tissue-adiponectin phenotypes

**Figure 4:**
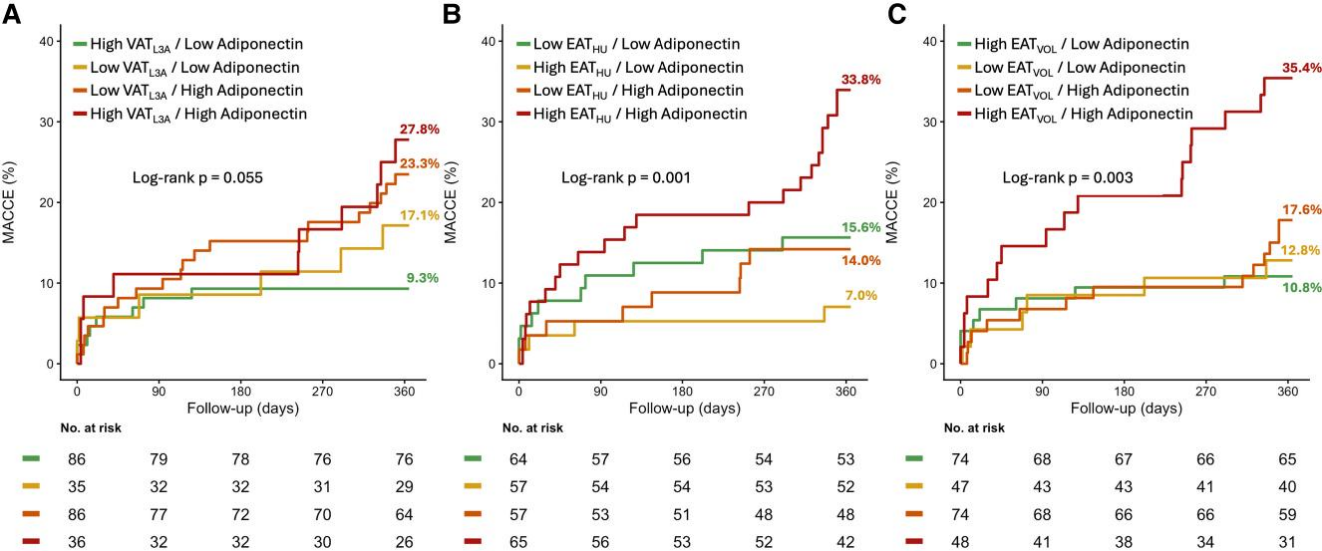
Kaplan–Meier curves for 1-year MACCE after TAVI across combined adipose tissue-adiponectin phenotypes. Cumulative incidence of MACCE stratified by median-based combinations of adiponectin with (*A*) VAT_L3A_, (*B*) EAT_HU_, and (*C*) EAT_VOL_. In each panel, curves from lowest to highest event rate are shown in green (pre-specified reference), gold, orange, and dark red. Numbers at risk shown below. EAT, epicardial adipose tissue; HU, Hounsfield units; L3A, lumbar vertebra 3 area; MACCE, major adverse cardiac and cerebrovascular events; TAVI, transcatheter aortic valve implantation; VAT, visceral adipose tissue.

Over a mean follow-up period of 344 ± 77 days, 44 patients (18%) experienced MACCE, including 25 deaths (10%), 12 HF hospitalizations (5%), and seven strokes (3%). Median-stratified adipose tissue-adiponectin phenotypes (VAT_L3A_, EAT_HU_, and EAT_VOL_) showed significant progressive risk gradients, with adjusted *P*-for-trend values of 0.003, 0.007, and 0.048, respectively (*Figure 4* and Supplementary material online, *Table S5*).

Both high VAT_L3A_/high adiponectin and low VAT_L3A_/high adiponectin groups were associated with increased risk with adjusted HRs of 3.68 (95% CI 1.44–9.44; *P* = 0.007) and 3.60 (95% CI 1.51–8.60; *P* = 0.004). Low VAT_L3A_/low adiponectin showed a trend towards higher risk (adjusted HR 2.84, 95% CI 0.95–8.46; *P* = 0.061), not reaching statistical significance. Event rates were 9.3, 17.1, 23.3, and 27.8% across the four groups.

Patients with high EAT_HU_/high adiponectin had the highest MACCE incidence (33.8% vs. 7.0, 14.0, and 15.6%) than the other groups. The adjusted HR for the high EAT_HU_/high adiponectin phenotype vs. the low EAT_HU_/low adiponectin reference was 2.05 (95% CI 0.94–4.50; *P* = 0.073). In contrast, high EAT_HU_/low adiponectin was associated with a numerically lower risk (adjusted HR 0.34, 95% CI 0.10–1.09; *P* = 0.069). However, these differences were not statistically significant.

Patients with high EAT_VOL_/high adiponectin had the highest MACCE incidence (35.4% vs. 10.8, 12.8, and 17.6%) and the strongest association with outcome (adjusted HR 4.25, 95% CI 1.80–10.07; *P* < 0.001) compared with the high EAT_VOL_/low adiponectin reference. Results of sensitivity analyses are presented in Supplementary material online, *Table S5*. After extended adjustment, only the high EAT_VOL_/high adiponectin group remained independently associated with MACCE.

## Discussion

This study demonstrates that quantitative and qualitative imaging of EAT and VAT characteristics, along with circulating adiponectin levels, provides complementary prognostic information for 1-year MACCE after TAVI. These findings support the conceptual value of integrating cardiometabolic markers with established risk factors for pre-procedural risk assessment in elderly TAVI candidates.

### Epicardial and visceral adiposity

Our results reinforce and extend previous observations that regional fat deposition, particularly EAT and VAT, is highly relevant to cardiovascular risk.^9,13^ While BMI is a crude measure of overall adiposity, it lacks specificity in capturing the regional distribution of inflammatory activity and metabolic phenotype.^12,31,32^ The so-called ‘obesity paradox’ reported in TAVI populations, where overweight and obese patients may experience better outcomes, demonstrates the limitations of BMI and the need for more nuanced markers.

We found that EAT_VOL_ correlated strongly with VAT_L3A_, indicating a shared pathophysiological pattern of central adiposity. However, VAT_L3A_ was not associated with EAT_HU_, underscoring the unique metabolic profile of EAT. EAT is a metabolically distinct organ with dual protective and pro-inflammatory biology that varies with anatomical location and stage of cardiac remodelling.^13^ In perivascular adipose tissue, inflammatory cytokines released by the inflamed vessel wall have been shown to suppress lipid accumulation in adjacent adipocytes, producing a detectable shift in CT attenuation,^33^ and in patients with atrial fibrillation, the degree of EAT fibrotic remodelling on histology has been shown to correlate directly with CT-measured EAT attenuation.^34^ In our TAVI cohort, however, EAT_HU_ and EAT_VOL_ did not independently predict 1-year MACCE when evaluated continuously. This is consistent with the findings of Donà and colleagues^35^, who applied the CRISP-CT methodology to 372 TAVI patients and observed no incremental prognostic value beyond established risk factors. Our findings demonstrated that the prognostic meaning of EAT_VOL_ emerges specifically when elevated adiponectin signalled active lipolytic and inflammatory biology, larger depots then represented proportionally greater myocardial exposure (adjusted HR 4.25).

The inverse association between VAT_L3A_ and 1-year MACCE (HR 0.64 per SD) aligns with findings from the same L3 AI-segmentation methodology in 866 TAVI patients^36^ and is quantitatively consistent with the U-shaped obesity paradox documented across 14 TAVI studies (*n* = 9692).^10^ Lower visceral fat in this age group reflects frailty, sarcopenia, or cardiac cachexia rather than healthy leanness. However, the continuous protective association of VAT_L3A_ was overridden in the combined phenotype analysis: patients with elevated adiponectin showed similarly high 1-year MACCE risk regardless of VAT_L3A_ status, indicating that once systemic neurohormonal stress is established, the protective effect of preserved visceral reserves is no longer detectable.

### The ‘adiponectin paradox’ in TAVI

Consistent with established physiology, adiponectin levels were inversely correlated with both EAT_VOL_ and VAT_L3A_, reflecting the known suppression of adiponectin by increased visceral adiposity.^37,38^ Interestingly, adiponectin showed a weak but statistically significant positive correlation with EAT_HU_, suggesting that adipose tissue quality and systemic metabolic stress may track together.

The ‘adiponectin paradox’ was also evident in our cohort. Although adiponectin is classically considered cardioprotective, higher circulating levels were associated with increased risk of MACCE, an observation in line with what has been reported in older adults and patients with chronic cardiac disease.^22–24^ The association between elevated circulating adiponectin and increased mortality in chronic HF was first described more than two decades ago, before CT-based adipose tissue imaging was available for routine cardiovascular risk stratification.^39^ A subsequent meta-analysis of seven studies comprising 862 HF patients confirmed that higher adiponectin approximately doubles the risk of all-cause mortality (relative risk 2.05, 95% CI 1.22–3.43).^40^ Our per-SD HR of 1.34 for MACCE falls within the expected range and is extended here to the TAVI population.

Adipocytes express the guanylyl cyclase-A receptor, and both atrial and B-type natriuretic peptides directly stimulate adiponectin secretion through this pathway.^41^ The positive correlations between adiponectin and both NT-proBNP and high-sensitivity cardiac troponin in our analysis are consistent with the clinical expression of this neurohormonal axis: patients with sufficient cardiac stress to elevate natriuretic peptides are those in whom the same peptides stimulate adipocyte adiponectin release. Large-scale Mendelian randomization analyses have found no causal effect of genetically determined adiponectin levels on major cardiovascular outcomes, indicating that circulating adiponectin functions primarily as a biomarker.^23^

Moreover, in healthy adults, direct adiponectin signalling predominates. In contrast, in elderly patients with established cardiovascular disease, peripheral adiponectin resistance, altered high-molecular-weight fractions, and non-adipocyte production together mean that circulating adiponectin rises precisely as its biological efficacy declines.^42–44^ A simultaneous rise in BNP and adiponectin therefore marks advanced cardiac dysfunction instead of effective compensation.

### Clinical implications

Our findings support a specific conceptual model for understanding adipose tissue biology in the TAVI population: circulating adiponectin and CT-derived fat parameters carry complementary information, and their combined evaluation reveals risk phenotypes. However, the clinical interpretation of pericardial fat imaging should not be uniform. A large EAT depot in a patient with low adiponectin carries different biological meaning from the same depot in a patient with elevated adiponectin, the former reflecting a metabolically quiet anatomy, the latter a substrate exposed to active neurohormonal drive. This dependence of local fat biology on the systemic environment is consistent with established mechanisms of adipose tissue natriuretic peptide-mediated lipolysis and adipokine regulation and provides a biological rationale for evaluating imaging and biomarker parameters together.

### Limitations

Several limitations should be acknowledged. First, the retrospective single-centre design and modest event count may limit the precision of subgroup analyses and the generalizability of the findings. Bootstrap internal validation confirmed minimal overfitting of the primary Cox models, with optimism-corrected C-indices of 0.61–0.68 indicating modest discrimination. Second, the relatively short follow-up period of 1 year may not capture the full spectrum of long-term outcomes related to adipose tissue biology. Third, while our AI-based imaging pipeline was validated internally, further external validation in a multicentre cohort is needed to ensure reproducibility across different imaging platforms and populations. In the present cohort, the visual quality-control review of the inferred pericardial masks was performed by a single trained operator. Formal multi-reader assessment of the accept/reject decision was not undertaken. Furthermore, fat-attenuation measurements are known to be influenced by technical CT acquisition and reconstruction parameters (tube voltage, reconstruction kernel, iterative-reconstruction strength, slice thickness, and contrast phase) as demonstrated in systematic phantom and clinical pericoronary fat studies.^45,46^ All CT acquisitions in the present study were performed on the same scanner using a standardized pre-TAVI protocol, which minimizes within-cohort variability from these factors. Cross-study comparability of absolute EAT_HU_ values should therefore be interpreted with caution.

## Conclusions

Circulating adiponectin and CT-derived VAT_L3A_ independently predicted 1-year MACCE after TAVI, while EAT parameters added prognostic information only when combined with adiponectin. Integrated adipose tissue imaging and biomarker phenotyping warrant prospective multicentre evaluation as a complement to conventional risk models in elderly TAVI candidates.

## Supplementary Material

oeag085_Supplementary_Data

## Data Availability

The data underlying this article will be shared on reasonable request to the corresponding author.
