# Supplementary material for: Imaging and biomarker-based risk stratification in TAVI: the role of epicardial fat, visceral fat, and adiponectin
Source: Eur Heart J Open. 2026 May 29;6(3):oeag085. doi: 10.1093/ehjopen/oeag085 (PMC13252506; doi:10.1093/ehjopen/oeag085)
Supplement: oeag085_Supplementary_Data [file oeag085_supplementary_data.docx]

Supplemental material

**Imaging and Biomarker-Based Risk Stratification in TAVI: The Role of Epicardial Fat, Visceral Fat, and Adiponectin**

Storozhenko et al.

**Address for Correspondence:**

Dr. Marc Vanderheyden

Cardiovascular Center Aalst, Moorselbaan 164, 9300, Aalst, Belgium

+32 53724439

[marc.vanderheyden@icloud.com](mailto:marc.vanderheyden@icloud.com)

**Table of contents**

[Supplemental Figure 1. Study flow chart. 3](#_Toc227585948)

[Supplemental Figure 2. Distribution of adipose tissue characteristics and adiponectin. 4](#_Toc227585949)

[Supplemental Figure 3. Correlation between adipose tissue characteristics and serum biomarkers. 5](#_Toc227585950)

[Supplemental Figure 4. Continuous risk profiling of adipose tissue characteristics and adiponectin for 1-year MACCE after TAVI using extended multivariable adjustment. 6](#_Toc227585951)

[Supplemental Figure 5. Association of adipose tissue characteristics and adiponectin with 1-year MACCE after TAVI according to quartiles using extended multivariable adjustment. 7](#_Toc227585952)

[Supplemental Table 1. Internal validation of Cox proportional hazards models for 1-year MACCE. 8](#_Toc227585953)

[Supplemental Table 2. Baseline characteristics stratified by combined VAT_L3A_ and adiponectin levels. 9](#_Toc227585954)

[Supplemental Table 3. Baseline characteristics stratified by combined EAT_HU_ and adiponectin levels. 11](#_Toc227585955)

[Supplemental Table 4. Baseline characteristics stratified by combined EAT_VOL_ and adiponectin levels. 13](#_Toc227585956)

[Supplemental Table 5. Risk stratification according to combined adipose tissue-adiponectin phenotypes. 15](#_Toc227585957)

# **Supplemental Figure 1.** Study flow chart.


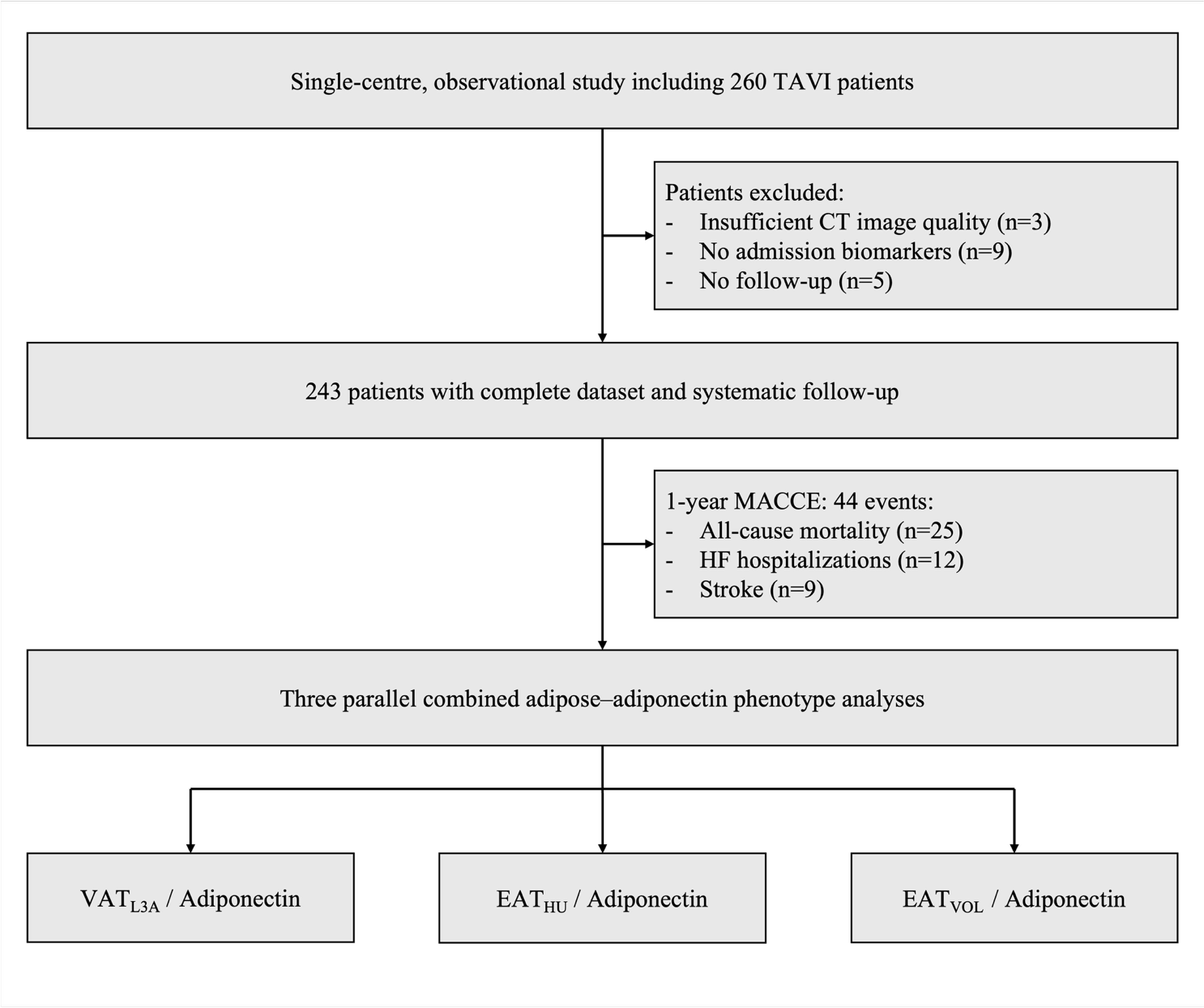


CT, computed tomography; EAT, epicardial adipose tissue; HU, Hounsfield units; TAVI, transcatheter aortic valve implantation.

# **Supplemental Figure 2.** Distribution of adipose tissue characteristics and adiponectin.


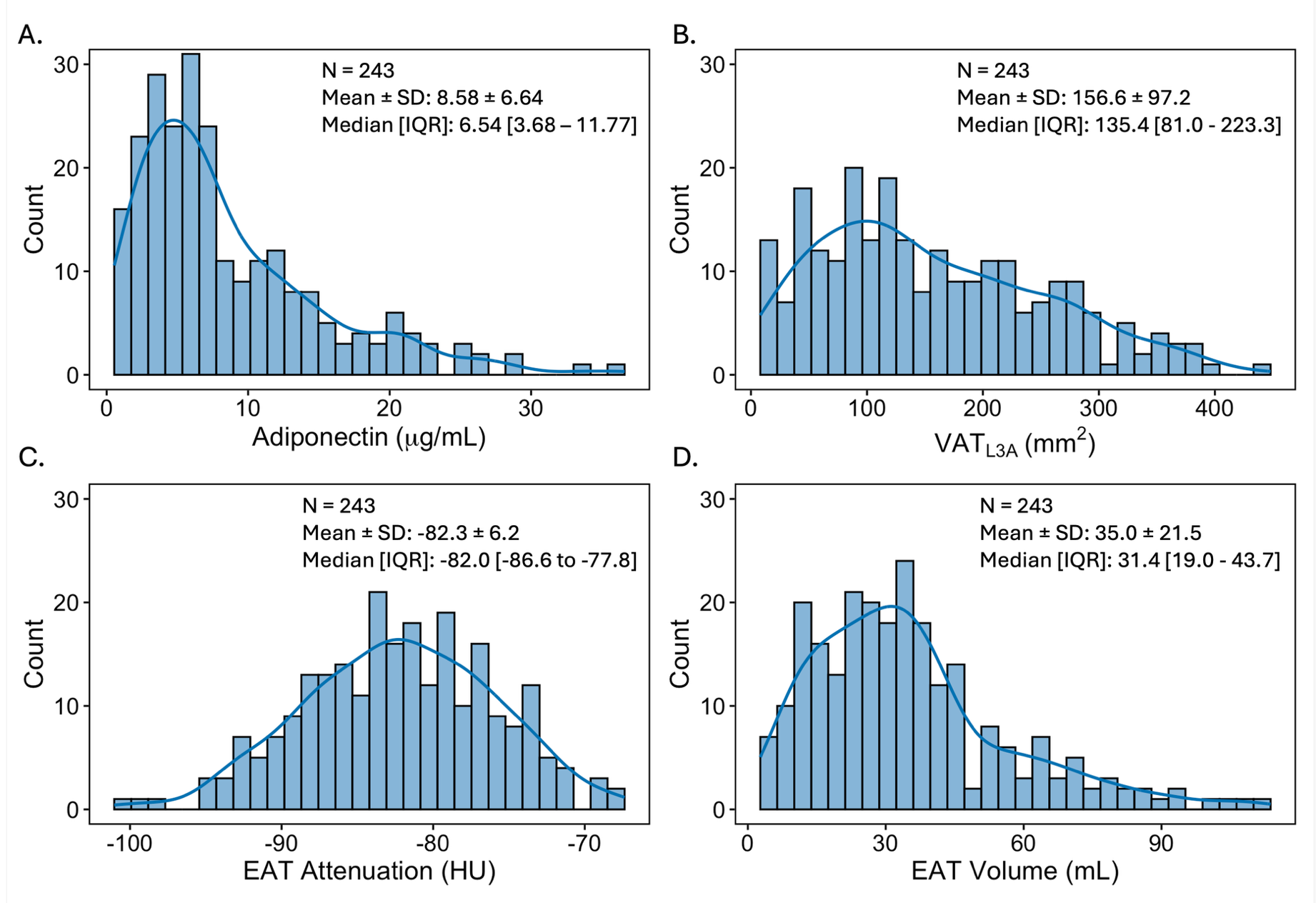


EAT, epicardial adipose tissue; HU, Hounsfield units; IQR, interquartile range; L3A, lumbar vertebra 3 area; SD, standard deviation; VAT, visceral adipose tissue.

# **Supplemental Figure 3.** Correlation between adipose tissue characteristics and serum biomarkers.


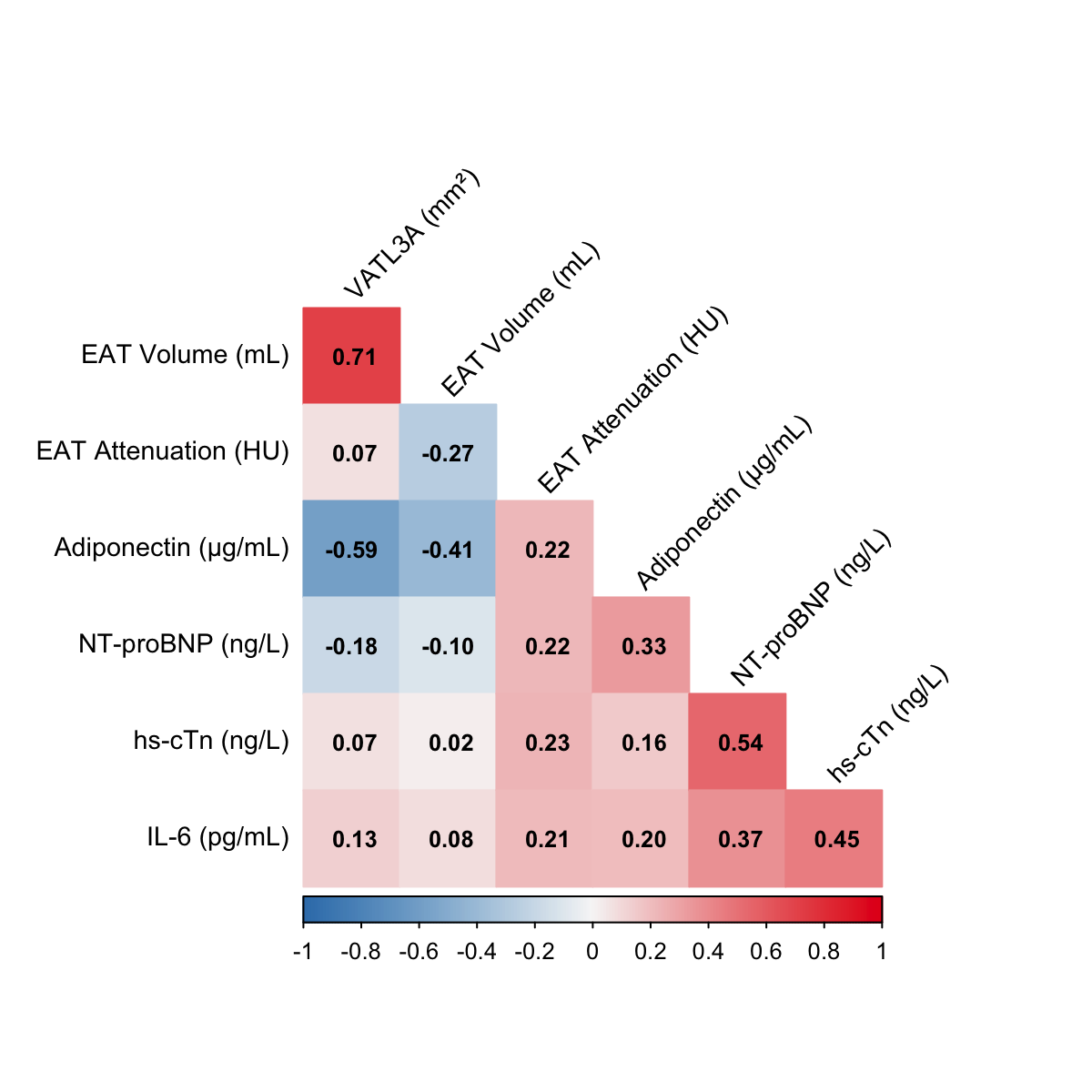


Spearman correlation matrix illustrates the associations between visceral adiposity, EAT parameters, circulating adiponectin, NT-proBNP, hs-cTn, and IL-6. Color shading indicates the magnitude and direction of correlation coefficients. EAT, epicardial adipose tissue; hs-cTn, high-sensitivity cardiac troponin; HU, Hounsfield units; IL‑6, interleukin‑6; L3A, lumbar vertebra 3 area; VAT, visceral adipose tissue.

#

# **Supplemental Figure 4.** Continuous risk profiling of adipose tissue characteristics and adiponectin for 1-year MACCE after TAVI using extended multivariable adjustment.


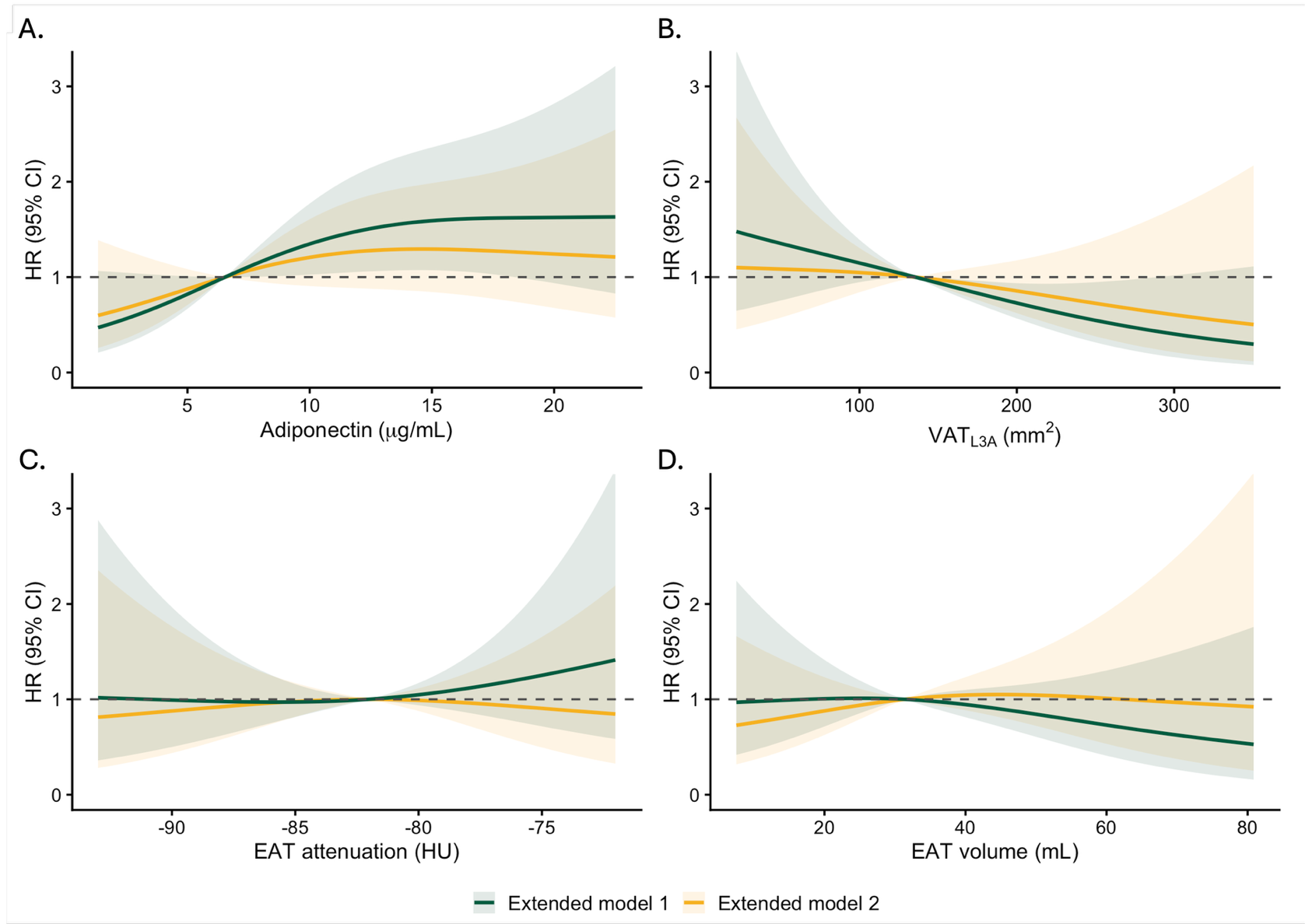


Extended model 1 included 243 patients and 44 MACCE events (Extended model 2: n = 239 due to missing covariate data). HRs are shown as solid lines with shaded areas representing 95% CI and are expressed relative to the median value of each variable. Extended model 1 (green) was adjusted for age, sex, STS score, frailty, renal function, atrial fibrillation, and left ventricular ejection fraction. Extended model 2 (orange) additionally included body mass index and NT-proBNP. CI, confidence interval; EAT, epicardial adipose tissue; HR, hazard ratio; HU, Hounsfield units; L3A, lumbar vertebra 3 area; MACCE, major adverse cardiac and cerebrovascular events; STS, Society of Thoracic Surgeons; TAVI, transcatheter aortic valve implantation; VAT, visceral adipose tissue.

# **Supplemental Figure 5.** Association of adipose tissue characteristics and adiponectin with 1-year MACCE after TAVI according to quartiles using extended multivariable adjustment.


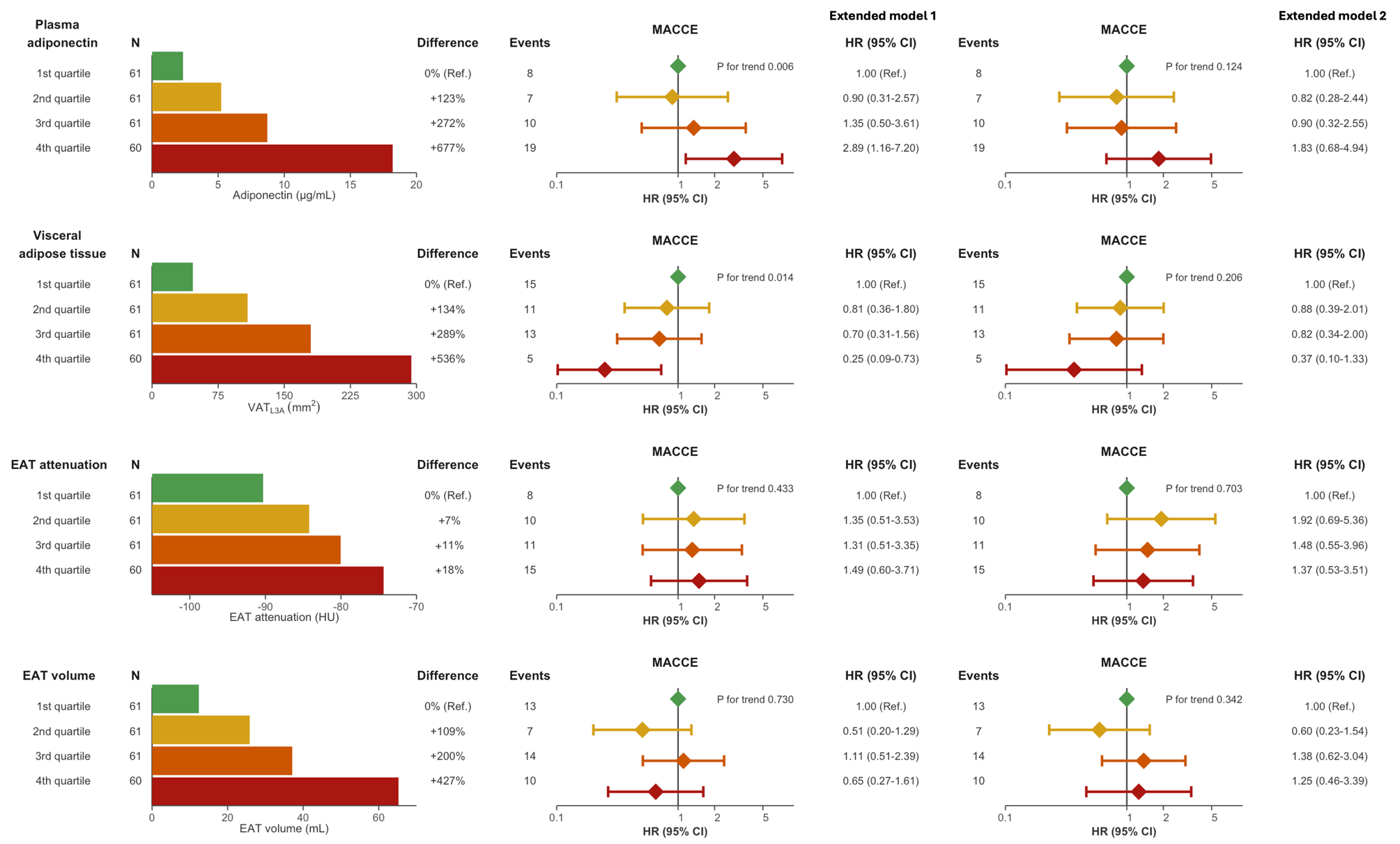


Mean values of plasma adiponectin, VAT_L3A_, EAT_HU_, and EAT_VOL_ across quartiles are shown as bars. Percentage differences relative to the first quartile are indicated. HRs with 95% confidence intervals for MACCE are indicated with diamonds and whiskers. Extended model 1 was adjusted for age, sex, STS score, frailty, renal function, atrial fibrillation, and left ventricular ejection fraction. Extended model 2 additionally included body mass index and NT-proBNP. CI, confidence interval; EAT, epicardial adipose tissue; HR, hazard ratio; HU, Hounsfield units; L3A, lumbar vertebra 3 area; MACCE, major adverse cardiac and cerebrovascular events; STS, Society of Thoracic Surgeons; TAVI, transcatheter aortic valve implantation; VAT, visceral adipose tissue.

# **Supplemental Table 1.** Internal validation of Cox proportional hazards models for 1-year MACCE.

| **Exposure** | **Model** | **Variables** | **EPV** | **Apparent**  **C-index** | **Optimism** | **Corrected**  **C-index** | **Calibration**  **slope** |
| --- | --- | --- | --- | --- | --- | --- | --- |
| Continuous biomarkers | | | | | | | |
| Adiponectin | Primary | 5 | 8.8 | 0.663 | 0.033 | 0.630 | 0.819 |
|  | Extended 1 | 8 | 5.5 | 0.676 | 0.051 | 0.625 | 0.721 |
|  | Extended 2 | 10 | 4.4 | 0.691 | 0.061 | 0.630 | 0.741 |
| VAT_L3A_ | Primary | 5 | 8.8 | 0.675 | 0.036 | 0.639 | 0.829 |
|  | Extended 1 | 8 | 5.5 | 0.686 | 0.052 | 0.634 | 0.728 |
|  | Extended 2 | 10 | 4.4 | 0.693 | 0.061 | 0.632 | 0.751 |
| EAT_HU_ | Primary | 5 | 8.8 | 0.649 | 0.038 | 0.610 | 0.781 |
|  | Extended 1 | 8 | 5.5 | 0.660 | 0.058 | 0.602 | 0.678 |
|  | Extended 2 | 10 | 4.4 | 0.689 | 0.064 | 0.624 | 0.742 |
| EAT_VOL_ | Primary | 5 | 8.8 | 0.649 | 0.037 | 0.612 | 0.790 |
|  | Extended 1 | 8 | 5.5 | 0.666 | 0.056 | 0.610 | 0.693 |
|  | Extended 2 | 10 | 4.4 | 0.691 | 0.063 | 0.628 | 0.740 |
| Combined phenotype groups | | | | | | | |
| VAT_L3A_ / Adiponectin | Primary | 7 | 6.3 | 0.693 | 0.045 | 0.648 | 0.784 |
|  | Extended 1 | 10 | 4.4 | 0.696 | 0.059 | 0.637 | 0.728 |
|  | Extended 2 | 12 | 3.7 | 0.707 | 0.071 | 0.636 | 0.723 |
| EAT_HU_ / Adiponectin | Primary | 7 | 6.3 | 0.721 | 0.038 | 0.684 | 0.821 |
|  | Extended 1 | 10 | 4.4 | 0.713 | 0.053 | 0.659 | 0.754 |
|  | Extended 2 | 12 | 3.7 | 0.745 | 0.067 | 0.678 | 0.726 |
| EAT_VOL_ / Adiponectin | Primary | 7 | 6.3 | 0.695 | 0.040 | 0.655 | 0.823 |
|  | Extended 1 | 10 | 4.4 | 0.696 | 0.054 | 0.642 | 0.766 |
|  | Extended 2 | 12 | 3.7 | 0.713 | 0.067 | 0.646 | 0.740 |

All models included 243 patients and 44 MACCE events (Extended model 2: n = 239 due to missing covariate data). The primary model was adjusted for age, sex, STS score, frailty. Extended model 1 was additionally adjusted for age, sex, STS score, frailty, renal function, atrial fibrillation, and left ventricular ejection fraction. Extended model 2 further included body mass index and NT-proBNP. Bootstrap internal validation (200 resamples) using Harrell’s optimism-correction method. EPV, events per variable; STS, Society of Thoracic Surgeons.

# **Supplemental Table 2.** Baseline characteristics stratified by combined VAT_L3A_ and adiponectin levels.

|  | **Overall**  **(N=243)** | **High VAT_L3A_**  **Low Adiponectin**  **(N=86)** | **Low VAT_L3A_**  **Low Adiponectin**  **(N=35)** | **Low VAT_L3A_**  **High Adiponectin**  **(N=86)** | **High VAT_L3A_**  **High Adiponectin**  **(N=36)** | **p-value** |
| --- | --- | --- | --- | --- | --- | --- |
| Clinical characteristics | | | | | | |
| Age, years | 81.9 ± 6.2 | 80.2 ± 5.9 | 81.9 ± 5.5 | 83.9 ± 6.4 | 81.4 ± 6.1 | 0.001 |
| Female sex | 132 (54.3) | 29 (33.7) | 26 (74.3) | 60 (71.4) | 17 (47.2) | <0.001 |
| BMI, kg/m^2^ | 25.0 [23.0-28.7] | 27.8 [25.1-32.5] | 24.0 [21.9-25.3] | 23.1 [20.8-24.9] | 26.2 [24.0-30.4] | <0.001 |
| Diabetes mellitus | 75 (30.9) | 38 (44.2) | 10 (28.6) | 11 (13.1) | 16 (44.4) | <0.001 |
| Hypertension | 174 (71.6) | 71 (83.5) | 26 (74.3) | 49 (58.3) | 28 (77.8) | 0.003 |
| Dyslipidemia | 197 (81.1) | 79 (92.9) | 30 (85.7) | 62 (73.8) | 26 (72.2) | 0.004 |
| COPD | 22 (9.0) | 8 (9.3) | 2 (5.7) | 9 (10.6) | 3 (8.3) | 0.863 |
| CAD | 98 (40.3) | 47 (54.7) | 7 (20.0) | 33 (39.3) | 11 (30.6) | 0.002 |
| PAD | 53 (21.8) | 21 (24.4) | 9 (25.7) | 18 (21.4) | 5 (13.9) | 0.578 |
| Prior PCI | 62 (25.5) | 27 (31.4) | 6 (17.1) | 23 (27.4) | 6 (17.1) | 0.235 |
| Prior CABG | 27 (11.1) | 10 (11.6) | 3 (8.6) | 9 (10.7) | 5 (13.9) | 0.910 |
| Prior myocardial infarction | 23 (9.6) | 12 (14.1) | 1 (2.9) | 7 (8.4) | 3 (8.6) | 0.264 |
| Pre-existing atrial fibrillation | 101 (41.5) | 33 (38.4) | 13 (38.2) | 37 (44.0) | 18 (50.0) | 0.626 |
| Pre-TAVI pacemaker or ICD | 3 (1.2) | 0 (0.0) | 0 (0.0) | 0 (0.0) | 3 (8.3) | 0.001 |
| Prior cerebrovascular accident | 36 (14.8) | 15 (17.6) | 5 (15.2) | 11 (13.3) | 5 (13.9) | 0.876 |
| NYHA functional class ≥ II | 233 (95.8) | 81 (94.2) | 33 (94.3) | 83 (96.5) | 36 (100.0) | 0.478 |
| Angina | 64 (26.3) | 33 (38.8) | 9 (25.7) | 13 (15.5) | 9 (25.7) | 0.008 |
| Frailty score | 4.0 [4.0-5.0] | 4.0 [4.0-6.0] | 4.0 [3.0-4.2] | 4.0 [3.7-5.0] | 5.0 [4.0-5.75] | 0.106 |
| STS score | 4.3 [2.8-7.0] | 3.9 [2.2-6.5] | 3.5 [2.8-6.2] | 4.5 [3.2-8.7] | 4.8 [3.6-6.7] | 0.038 |
| EuroSCORE II | 4.0 [2.6-7.3] | 3.7 [2.2-6.8] | 3.7 [2.1-5.4] | 4.7 [3.1-8.2] | 5.4 [2.4-8.8] | 0.060 |
| Creatinine, mg/dL | 1.0 [0.85-1.4] | 1.1 [0.90-1.5] | 1.0 [0.66-1.2] | 0.96 [0.79-1.3] | 1.0 [0.76-1.8] | 0.019 |
| NT-proBNP, ng/L | 1366 [572-2546] | 1129 [470-1767] | 928 [401-2000] | 1795 [877-3385] | 2037 [968-7428] | 0.001 |
| hs-cTn, ng/L | 25 [16-53] | 22 [17-39] | 19 [14-33] | 23 [16-49] | 53 [19-94] | 0.038 |
| Adiponectin, µg/mL | 6.54 [3.68-11.77] | 3.41 [2.19-4.83] | 4.55 [3.42-5.74] | 12.10 [9.23-18.55] | 9.71 [7.45-13.24] | <0.001 |
| Echocardiographic and CT characteristics | | | | | | |
| LVEF, % | 55.4 ± 16.0 | 54.5 ± 14.0 | 59.6 ± 19.3 | 56.4 ± 15.7 | 52.5 ± 15.8 | 0.344 |
| AV peak velocity, m/s | 4.0 ± 0.8 | 3.9 ± 0.7 | 4.2 ± 0.7 | 4.0 ± 0.9 | 4.1 ± 1.0 | 0.807 |
| AV mean gradient, mmHg | 44.2 ± 17.3 | 42.8 ± 16.0 | 46.3 ± 16.0 | 44.0 ± 18.2 | 47.0 ± 20.6 | 0.759 |
| AVA, cm^2^ | 0.75 ± 0.31 | 0.80 ± 0.23 | 0.69 ± 0.21 | 0.71 ± 0.38 | 0.76 ± 0.33 | 0.403 |
| AVA index, cm^2^/m^2^ | 0.42 ± 0.18 | 0.42 ± 0.11 | 0.42 ± 0.12 | 0.47 ± 0.24 | 0.40 ± 0.15 | 0.493 |
| EAT_HU_, HU | -82.0 [-86.6 to -77.7] | -82.3 [-86.3 to -78.8] | -83.1 [-90.4 to -79.1] | -81.6 [-85.6 to -75.1] | -81.8 [-86.0 to -79.5] | 0.040 |
| EAT_VOL_, mL | 31.4 [19.0-43.7] | 42.8 [30.9-64.2] | 27.2 [22.5-37.1] | 17.3 [11.5-31.0] | 38.6 [31.3-50.5] | <0.001 |
| VAT_L3A_, mm^2^ | 136.1 [81.3-223.9] | 236.4 [197.6-289.2] | 107.1 [83.6-119.7] | 67.1 [42.0-93.8] | 186.9 [163.5-242.1] | <0.001 |
| Procedural characteristics | | | | | | |
| Transfemoral access | 243 (100.0) | 86 (100.0) | 35 (100.0) | 86 (100.0) | 36 (100.0) |  |
| Valve-in-valve | 12 (4.9) | 3 (3.5) | 0 (0.0) | 8 (9.5) | 1 (2.8) | 0.099 |
| Device type |  |  |  |  |  | 0.737 |
| Self-expanding | 236 (97.1) | 83 (96.5) | 35 (100.0) | 83 (96.5) | 35 (97.2) |  |
| Balloon expandable | 7 (2.8) | 3 (3.5) | 0 (0.0) | 3 (3.5) | 1 (2.8) |  |
| Vascular complications |  |  |  |  |  | 0.747 |
| Minor | 17 (6.9) | 4 (4.7) | 2 (5.7) | 9 (10.5) | 2 (5.6) |  |
| Major | 5 (2.0) | 2 (2.3) | 1 (2.9) | 2 (2.3) | 0 (0.0) |  |
| Cerebrovascular accident | 9 (3.7) | 0 (0.0) | 2 (5.7) | 5 (6.0) | 2 (5.6) | 0.158 |
| Myocardial infarction | 1 (0.4) | 0 (0.0) | 0 (0.0) | 1 (1.2) | 0 (0.0) | 0.598 |
| Bleeding complications | 13 (5.3) | 1 (2.9) | 4 (4.8) | 2 (5.6) | 0.818 | 1 (2.9) |
| Permanent pacemaker implantation | 57 (23.4) | 27 (31.4) | 7 (20.0) | 15 (17.9) | 8 (22.2) | 0.194 |
| Acute kidney injury | 6 (2.5) | 2 (2.3) | 1 (2.9) | 3 (3.6) | 0 (0.0) | 0.717 |

Continuous variables are presented as mean ± SD or median [IQR], when appropriate; categorical data as n (%).

AV, aortic valve; AVA, aortic valve area; BMI, body mass index; CABG, coronary artery bypass graft; CAD, coronary artery disease; COPD, chronic obstructive pulmonary disease; CT, computed tomography; EAT, epicardial adipose tissue; hs-cTn, high-sensitivity cardiac troponin; HU, Hounsfield units; ICD, implantable cardioverter-defibrillator; LVEF, left ventricular ejection fraction; L3A, lumbar vertebra 3 area; NYHA, New York Heart Association; PAD, peripheral arterial disease; PCI, percutaneous coronary intervention; STS, Society of Thoracic Surgeons; TAVI, transcatheter aortic valve implantation; VAT, visceral adipose tissue.

# **Supplemental Table 3.** Baseline characteristics stratified by combined EAT_HU_ and adiponectin levels.

|  | **Overall**  **(N=243)** | **Low EAT_HU_**  **Low Adiponectin**  **(N=64)** | **High EAT_HU_**  **Low Adiponectin**  **(N=57)** | **Low EAT_HU_**  **High Adiponectin**  **(N=57)** | **High EAT_HU_**  **High Adiponectin**  **(N=65)** | **p-value** |
| --- | --- | --- | --- | --- | --- | --- |
| Clinical characteristics | | | | | | |
| Age, years | 81.9 ± 6.2 | 79.9 ± 6.0 | 81.6 ± 5.64 | 83.6 ± 5.4 | 82.8 ± 7.12 | 0.007 |
| Female sex | 132 (54.3) | 36 (56.2) | 19 (33.3) | 42 (75.0) | 35 (54.7) | <0.001 |
| BMI, kg/m^2^ | 25.0 [23.0-28.7] | 27.4 [23.8-32.2] | 26.1 [24.6-28.5] | 23.8 [20.8-26.3] | 24.0 [21.8-26.6] | <0.001 |
| Diabetes mellitus | 75 (30.9) | 27 (42.2) | 21 (36.8) | 12 (21.4) | 15 (23.4) | 0.033 |
| Hypertension | 174 (71.6) | 50 (78.1) | 47 (83.9) | 34 (60.7) | 43 (67.2) | 0.023 |
| Dyslipidemia | 197 (81.1) | 59 (92.2) | 50 (89.3) | 43 (76.8) | 45 (70.3) | 0.004 |
| COPD | 22 (9.0) | 5 (7.8) | 5 (8.8) | 5 (8.9) | 7 (10.8) | 0.949 |
| CAD | 98 (40.3) | 28 (43.8) | 26 (45.6) | 23 (41.1) | 21 (32.8) | 0.481 |
| PAD | 53 (21.8) | 14 (21.9) | 16 (28.1) | 12 (21.4) | 11 (17.2) | 0.552 |
| Prior PCI | 62 (25.5) | 17 (26.6) | 16 (28.1) | 17 (30.4) | 12 (19.0) | 0.517 |
| Prior CABG | 27 (11.1) | 6 (9.4) | 7 (12.3) | 3 (5.4) | 11 (17.2) | 0.211 |
| Prior myocardial infarction | 23 (9.6) | 4 (6.2) | 9 (16.1) | 6 (10.9) | 4 (6.3) | 0.223 |
| Pre-existing atrial fibrillation | 101 (41.5) | 29 (46.0) | 17 (29.8) | 23 (41.1) | 32 (50.0) | 0.134 |
| Pre-TAVI pacemaker or ICD | 3 (1.2) | 0 (0.0) | 0 (0.0) | 1 (1.8) | 2 (3.1) | 0.321 |
| Prior cerebrovascular accident | 36 (14.8) | 7 (11.1) | 13 (23.6) | 7 (12.5) | 9 (14.3) | 0.239 |
| NYHA functional class ≥ II | 233 (95.8) | 60 (93.8) | 54 (94.7) | 55 (96.5) | 64 (98.5) | 0.557 |
| Angina | 64 (26.3) | 24 (37.5) | 18 (32.1) | 12 (21.8) | 10 (15.6) | 0.025 |
| Frailty score | 4.0 [4.0-5.0] | 4.0 [4.0-5.0] | 4.0 [4.0-6.0] | 4.0 [4.0-5.0] | 4.0 [4.0-6.0] | 0.872 |
| STS score | 4.3 [2.8-7.0] | 3.4 [2.5-5.4] | 4.0 [2.4-7.2] | 4.4 [3.1-7.7] | 5.2 [3.6-8.3] | 0.011 |
| EuroSCORE II | 4.0 [2.6-7.3] | 3.5 [2.0-6.4] | 3.9 [2.5-6.2] | 3.8 [2.6-6.8] | 5.6 [3.3-11.4] | 0.012 |
| Creatinine, mg/dL | 1.0 [0.85-1.4] | 1.0 [0.89-1.3] | 1.2 [0.86-1.4] | 0.97 [0.76-1.4] | 0.99 [0.80-1.4] | 0.471 |
| NT-proBNP, ng/L | 1366 [572-2546] | 1074 [356-1609] | 1207 [592-2382] | 1441 [573- 2719] | 2458 [1161-4674] | 0.001 |
| hs-cTn, ng/L | 25 [16-53] | 21 [15-42] | 26 [20-37] | 21 [15-42] | 35 [20-108] | 0.012 |
| Adiponectin, µg/mL | 6.54 [3.68-11.77] | 3.72 [2.41-5.46] | 3610 [2.23- 5.11] | 11.05 [7.73-16.23] | 11.99 [8.89-15.87] | <0.001 |
| Echocardiographic and CT characteristics | | | | | | |
| LVEF, % | 55.4 ± 16.0 | 55.7 ± 15.2 | 56.0 ± 16.6 | 59.8 ± 14.5 | 51.3 ± 15.9 | 0.042 |
| AV peak velocity, m/s | 4.0 ± 0.8 | 3.9 ± 0.6 | 4.1 ± 0.9 | 4.0 ± 1.1 | 4.1 ± 0.8 | 0.723 |
| AV mean gradient, mmHg | 44.2 ± 17.3 | 41.7 ± 14.4 | 46.3 ± 17.9 | 44.6 ± 20.7 | 45.3 ± 16.9 | 0.699 |
| AVA, cm^2^ | 0.75 ± 0.31 | 0.79 ± 0.25 | 0.74 ± 0.20 | 0.69 ± 0.36 | 0.76 ± 0.38 | 0.571 |
| AVA index, cm^2^/m^2^ | 0.42 ± 0.18 | 0.44 ± 0.12 | 0.38 ± 0.09 | 0.42 ± 0.19 | 0.48 ± 0.24 | 0.231 |
| EAT_HU_, HU | -82.0 [-86.6 to -77.7] | -86.9 [-90.5 to -83.9] | -78.9 [-80.8 to -77.1] | -86.4 [-88.7 to -84.0] | -76.1 [-79.3 to -73.7] | <0.001 |
| EAT_VOL_, mL | 31.4 [19.0-43.7] | 43.6 [27.9-64.8] | 33.0 [26.0-41.9] | 29.4 [17.6-39.8] | 20.8 [12.7-33.5] | <0.001 |
| VAT_L3A_, mm^2^ | 136.1 [81.3-223.9] | 205.9 [121.9-276.3] | 199.7 [128.8-267.0] | 91.8 [44.9-150.3] | 92.8 [51.7-144.5] | <0.001 |
| Procedural characteristics | | | | | | |
| Transfemoral access | 243 (100.0) | 64 (100.0) | 57 (100.0) | 57 (100.0) | 65 (100.0) |  |
| Valve-in-valve | 12 (4.9) | 2 (3.1) | 1 (1.8) | 3 (5.4) | 6 (9.4) | 0.226 |
| Device type |  |  |  |  |  | 0.470 |
| Self-expanding | 236 (97.1) | 61 (95.3) | 57 (100.0) | 55 (96.5) | 63 (96.9) |  |
| Balloon expandable | 7 (2.8) | 3 (4.7) | 0 (0.0) | 2 (3.5) | 2 (3.1) |  |
| Vascular complications |  |  |  |  |  | 0.594 |
| Minor | 17 (6.9) | 3 (4.7) | 3 (5.3) | 3 (5.3) | 8 (12.3) |  |
| Major | 5 (2.0) | 1 (1.6) | 2 (3.5) | 1 (1.8) | 1 (1.5) |  |
| Cerebrovascular accident | 9 (3.7) | 2 (3.1) | 0 (0.0) | 2 (3.6) | 5 (7.8) | 0.155 |
| Myocardial infarction | 1 (0.4) | 0 (0.0) | 0 (0.0) | 1 (1.8) | 0 (0.0) | 0.345 |
| Bleeding complications | 13 (5.3) | 2 (3.1) | 5 (8.8) | 4 (7.1) | 2 (3.1) | 0.407 |
| Permanent pacemaker implantation | 57 (23.4) | 18 (28.1) | 16 (28.1) | 12 (21.4) | 11 (17.2) | 0.398 |
| Acute kidney injury | 6 (2.5) | 2 (3.1) | 1 (1.8) | 1 (1.8) | 2 (3.1) | 0.929 |

Continuous variables are presented as mean ± SD or median [IQR], when appropriate; categorical data as n (%).

AV, aortic valve; AVA, aortic valve area; BMI, body mass index; CABG, coronary artery bypass graft; CAD, coronary artery disease; COPD, chronic obstructive pulmonary disease; CT, computed tomography; EAT, epicardial adipose tissue; hs-cTn, high-sensitivity cardiac troponin; HU, Hounsfield units; ICD, implantable cardioverter-defibrillator; LVEF, left ventricular ejection fraction; L3A, lumbar vertebra 3 area; NYHA, New York Heart Association; PAD, peripheral arterial disease; PCI, percutaneous coronary intervention; STS, Society of Thoracic Surgeons; TAVI, transcatheter aortic valve implantation; VAT, visceral adipose tissue.

# **Supplemental Table 4.** Baseline characteristics stratified by combined EAT_VOL_ and adiponectin levels.

|  | **Overall**  **(N=243)** | **High EAT_VOL_**  **Low Adiponectin**  **(N=74)** | **Low EAT_VOL_**  **Low Adiponectin**  **(N=47)** | **Low EAT_VOL_**  **High Adiponectin**  **(N=74)** | **High EAT_VOL_**  **High Adiponectin**  **(N=48)** | **p-value** |
| --- | --- | --- | --- | --- | --- | --- |
| Clinical characteristics | | | | | | |
| Age, years | 81.9 ± 6.2 | 80.0 ± 5.6 | 81.7 ± 6.1 | 83.8 ± 5.4 | 82.1 ± 7.5 | 0.003 |
| Female sex | 132 (54.3) | 27 (36.5) | 28 (59.6) | 51 (70.8) | 26 (54.2) | <0.001 |
| BMI, kg/m^2^ | 25.0 [23.0-28.7] | 27.8 [25.0-31.6] | 24.7 [22.2-27.0] | 23.6 [21.0-25.2] | 24.1 [22.5-29.3] | <0.001 |
| Diabetes mellitus | 75 (30.9) | 33 (44.6) | 15 (31.9) | 12 (16.7) | 15 (31.2) | 0.004 |
| Hypertension | 174 (71.6) | 62 (83.8) | 35 (76.1) | 41 (56.9) | 36 (75.0) | 0.003 |
| Dyslipidemia | 197 (81.1) | 68 (91.9) | 41 (89.1) | 51 (70.8) | 37 (77.1) | 0.004 |
| COPD | 22 (9.0) | 9 (12.2) | 1 (2.1) | 6 (8.2) | 6 (12.5) | 0.227 |
| CAD | 98 (40.3) | 38 (51.4) | 16 (34.0) | 26 (36.1) | 18 (37.5) | 0.159 |
| PAD | 53 (21.8) | 14 (18.9) | 16 (34.0) | 15 (20.8) | 8 (16.7) | 0.155 |
| Prior PCI | 62 (25.5) | 25 (33.8) | 8 (17.0) | 17 (23.6) | 12 (25.5) | 0.209 |
| Prior CABG | 27 (11.1) | 6 (8.1) | 7 (14.9) | 8 (11.1) | 6 (12.5) | 0.697 |
| Prior myocardial infarction | 23 (9.6) | 13 (17.6) | 0 (0.0) | 3 (4.2) | 7 (14.9) | 0.003 |
| Pre-existing atrial fibrillation | 101 (41.5) | 32 (43.2) | 14 (30.4) | 31 (43.1) | 24 (50.0) | 0.277 |
| Pre-TAVI pacemaker or ICD | 3 (1.2) | 0 (0.0) | 0 (0.0) | 0 (0.0) | 3 (6.2) | 0.007 |
| Prior cerebrovascular accident | 36 (14.8) | 8 (11.1) | 12 (26.1) | 13 (18.1) | 3 (6.4) | 0.037 |
| NYHA functional class ≥ II | 233 (95.8) | 71 (95.9) | 43 (91.5) | 71 (95.9) | 48 (100.0) | 0.225 |
| Angina | 64 (26.3) | 27 (36.5) | 15 (32.6) | 10 (13.9) | 12 (25.5) | 0.015 |
| Frailty score | 4.0 [4.0-5.0] | 4.0 [4.0-6.0] | 4.0 [4.0-5.0] | 4.0 [3.0-5.5] | 4.0 [4.0-5.0] | 0.716 |
| STS score | 4.3 [2.8-7.0] | 3.3 [2.2-5.6] | 4.4 [2.9-7.4] | 4.3 [2.9-7.5] | 5.3 [3.9-8.1] | 0.002 |
| EuroSCORE II | 4.0 [2.6-7.3] | 3.1 [1.9-5.3] | 4.8 [3.1-7.4] | 5.1 [3.0-10.2] | 4.6 [2.9-7.2] | 0.006 |
| Creatinine, mg/dL | 1.0 [0.85-1.4] | 1.0 [0.86-1.5] | 1.1 [0.90-1.2] | 1.0 [0.83-1.4] | 0.96 [0.74-1.5] | 0.684 |
| NT-proBNP, ng/L | 1366 [572-2546] | 1024 [413-1573] | 1219 [577-2264] | 2364 [865-4655] | 1393 [956-3601] | <0.001 |
| hs-cTn, ng/L | 25 [16-53] | 22 [16-34] | 27 [16-39] | 24 [16-59] | 34 [17-60] | 0.351 |
| Adiponectin, µg/mL | 6.54 [3.68-11.77] | 3.68 [2.59-5.39] | 3.64 [2.24-4.89] | 11.82 [8.72-18.95] | 10.89 [7.92-14.46] | <0.001 |
| Echocardiographic and CT characteristics | | | | | | |
| LVEF, % | 55.4 ± 16.0 | 56.1 ± 13.9 | 55.4 ± 18.2 | 56.9 ± 16.1 | 52.8 ± 14.9 | 0.591 |
| AV peak velocity, m/s | 4.0 ± 0.8 | 3.9 ± 0.7 | 4.1 ± 0.8 | 4.1 ± 0.8 | 3.9 ± 1.1 | 0.711 |
| AV mean gradient, mmHg | 44.2 ± 17.3 | 42.4 ± 15.5 | 45.6 ± 16.8 | 45.6 ±17.5 | 43.9 ± 21.0 | 0.807 |
| AVA, cm^2^ | 0.75 ± 0.31 | 0.79 ± 0.23 | 0.75 ± 0.24 | 0.69 ± 0.35 | 0.78 ± 0.39 | 0.502 |
| AVA index, cm^2^/m^2^ | 0.42 ± 0.18 | 0.42 ± 0.11 | 0.42 ± 0.11 | 0.44 ± 0.22 | 0.46 ± 0.23 | 0.833 |
| EAT_HU_, HU | -82.0 [-86.6 to -77.7] | -83.2 [-87.3 to -80.4] | -81.6 [-86.8 to -77.3] | -79.8 [-85.1 to -75.1] | -83.2 [-86.4 to -79.2] | 0.008 |
| EAT_VOL_, mL | 31.4 [19.0-43.7] | 52.2 [38.7-66.9] | 24.7 [18.9-28.1] | 15.0 [10.8-22.1] | 39.8 [34.5-46.3] | <0.001 |
| VAT_L3A_, mm^2^ | 136.1 [81.3-223.9] | 226.7 [164.6-284.2] | 139.2 [108.3-217.5] | 65.8 [36.9-107.1] | 144.78 [93.8-212.5] | <0.001 |
| Procedural characteristics | | | | | | |
| Transfemoral access | 243 (100.0) | 74 (100.0) | 47 (100.0) | 74 (100.0) | 48 (100.0) |  |
| Valve-in-valve | 12 (4.9) | 2 (2.7) | 1 (2.1) | 6 (8.3) | 3 (6.2) | 0.322 |
| Device type |  |  |  |  |  | 0.562 |
| Self-expanding | 236 (97.1) | 71 (95.9) | 47 (100.0) | 72 (97.3) | 46 (95.8) |  |
| Balloon expandable | 7 (2.8) | 3 (4.1) | 0 (0.0) | 2 (2.7) | 2 (4.2) |  |
| Vascular complications |  |  |  |  |  | 0.509 |
| Minor | 17 (6.9) | 4 (5.4) | 2 (4.3) | 7 (9.5) | 4 (8.3) |  |
| Major | 5 (2.0) | 3 (4.1) | 0 (0.0) | 2 (2.7) | 0 (0.0) |  |
| Cerebrovascular accident | 9 (3.7) | 0 (0.0) | 2 (4.3) | 3 (4.2) | 4 (8.3) | 0.123 |
| Myocardial infarction | 1 (0.4) | 0 (0.0) | 0 (0.0) | 1 (1.4) | 0 (0.0) | 0.502 |
| Bleeding complications | 13 (5.3) | 4 (5.4) | 3 (6.4) | 1 (1.4) | 5 (10.4) | 0.193 |
| Permanent pacemaker implantation | 57 (23.4) | 21 (28.4) | 13 (27.7) | 13 (18.1) | 10 (20.8) | 0.425 |
| Acute kidney injury | 6 (2.5) | 0 (0.0) | 3 (6.4) | 2 (2.8) | 1 (2.1) | 0.181 |

Continuous variables are presented as mean ± SD or median [IQR], when appropriate; categorical data as n (%).

AV, aortic valve; AVA, aortic valve area; BMI, body mass index; CABG, coronary artery bypass graft; CAD, coronary artery disease; COPD, chronic obstructive pulmonary disease; CT, computed tomography; EAT, epicardial adipose tissue; hs-cTn, high-sensitivity cardiac troponin; HU, Hounsfield units; ICD, implantable cardioverter-defibrillator; LVEF, left ventricular ejection fraction; L3A, lumbar vertebra 3 area; NYHA, New York Heart Association; PAD, peripheral arterial disease; PCI, percutaneous coronary intervention; STS, Society of Thoracic Surgeons; TAVI, transcatheter aortic valve implantation; VAT, visceral adipose tissue.

# **Supplemental Table 5.** Risk stratification according to combined adipose tissue-adiponectin phenotypes.

| **Group** | **N** | **Primary Model*** | | | | **Extended Model 1†** | | | **Extended Model 2‡** | | |
| --- | --- | --- | --- | --- | --- | --- | --- | --- | --- | --- | --- |
|  |  | **Events** | **Adjusted HR (95% CI)** | **p-value** | **p-trend** | **Adjusted HR (95% CI)** | **p-value** | **p-trend** | **Adjusted HR (95% CI)** | **p-value** | **p-trend** |
| High VAT_L3A_  Low Adiponectin | 86 | 8 | Reference | - | 0.003 | Reference | - | 0.005 | Reference | - | 0.138 |
| Low VAT_L3A_  Low Adiponectin | 35 | 6 | 2.84 (0.95-8.46) | 0.061 |  | 3.12 (1.02–9.54) | 0.046 |  | 1.95 (0.61–6.27) | 0.260 |  |
| Low VAT_L3A_  High Adiponectin | 86 | 20 | 3.60 (1.51-8.60) | 0.004 |  | 3.62 (1.47–8.89) | 0.005 |  | 2.15 (0.80–5.74) | 0.127 |  |
| High VAT_L3A_  High Adiponectin | 36 | 10 | 3.68 (1.44-9.44) | 0.007 |  | 3.45 (1.33–8.96) | 0.011 |  | 2.22 (0.81–6.06) | 0.119 |  |
| Low EAT_HU_  Low Adiponectin | 64 | 10 | Reference | - | 0.007 | Reference | - | 0.012 | Reference | - | 0.196 |
| High EAT_HU_  Low Adiponectin | 57 | 4 | 0.34 (0.10-1.09) | 0.069 |  | 0.32 (0.10–1.07) | 0.064 |  | 0.30 (0.09–1.01) | 0.051 |  |
| Low EAT_HU_  High Adiponectin | 57 | 8 | 0.95 (0.36-2.51) | 0.921 |  | 0.90 (0.34–2.37) | 0.831 |  | 0.62 (0.23–1.68) | 0.349 |  |
| High EAT_HU_  High Adiponectin | 65 | 22 | 2.05 (0.94-4.50) | 0.073 |  | 2.00 (0.90–4.46) | 0.090 |  | 1.24 (0.52–2.97) | 0.632 |  |
| High EAT_VOL_  Low Adiponectin | 74 | 8 | Reference | - | 0.048 | Reference | - | 0.067 | Reference | - | 0.616 |
| Low EAT_VOL_  Low Adiponectin | 47 | 6 | 1.34 (0.46-3.97) | 0.592 |  | 1.52 (0.51–4.53) | 0.454 |  | 1.11 (0.37–3.35) | 0.852 |  |
| Low EAT_VOL_  High Adiponectin | 74 | 13 | 1.93 (0.76-4.88) | 0.167 |  | 1.89 (0.73–4.86) | 0.188 |  | 1.06 (0.40–2.83) | 0.904 |  |
| High EAT_VOL_  High Adiponectin | 48 | 17 | 4.25 (1.80-10.07) | <0.001 |  | 4.18 (1.76–9.96) | 0.001 |  | 2.67 (1.09–6.56) | 0.032 |  |

*Adjusted for age, sex, STS score, and frailty.

†Primary Model plus renal function, atrial fibrillation, and left ventricular ejection fraction.

‡Extended Model 1 plus body mass index and NT-proBNP.

The risk of 1-year MACCE was estimated using the pre-specified low-risk group as the reference category for each combined phenotype, based on the patterns observed in continuous and quartile analyses.

CI, confidence interval; EAT, epicardial adipose tissue; HR, hazard ratio; HU, Hounsfield units; L3A, lumbar vertebra 3 area; MACCE, major adverse cardiac and cerebrovascular events; VAT, visceral adipose tissue.
